# Home-Based Cardiac Rehabilitation in Brazil’s Public Health Care: Protocol for a Randomized Controlled Trial

**DOI:** 10.2196/13901

**Published:** 2019-11-07

**Authors:** Ana Paula Lima, Isabella Oliveira Nascimento, Anne Caroline A Oliveira, Thiago Henrique S Martins, Danielle A Gomes Pereira, Raquel Rodrigues Britto

**Affiliations:** 1 Physical Therapy Department Universidade Federal de Minas Gerais Belo Horizonte Brazil

**Keywords:** cardiac rehabilitation, coronary disease, exercise

## Abstract

**Background:**

Coronary artery disease (CAD) is among the main causes of hospitalization and death worldwide, therefore, the implementation of programs to reduce its impact is necessary. Supervised cardiac rehabilitation has been shown to have positive effects on CAD control. However, there are barriers to patient participation in the traditional, face-to-face cardiac rehabilitation programs, mainly in low-resource environments.

**Objective:**

This study aimed to verify patient compliance to a home-based cardiac rehabilitation program, which includes unsupervised health education and physical exercises, guided by telephone. Moreover, we compare this new method to the traditional supervised cardiac rehabilitation offered in most hospital centers.

**Methods:**

We present here a two-arm, single-blinded, and randomized controlled design protocol, which compares the traditional cardiac rehabilitation (CenterRehab) with the home-based cardiac rehabilitation (Home-Based) in 72 patients affected by CAD. The primary outcome is the compliance to the cardiac rehabilitation sessions. The secondary outcomes (to evaluate effectiveness) include measurable variables such as functional capacity, CAD risk factors (blood pressure, waist circumference, glycemic, cholesterol levels, depressive symptoms, and the level of physical activity), the patient’s quality of life, the disease knowledge, and the morbidity rate. Parameters such as the program cost and the usability will also be evaluated. The programs will last 12 weeks, with a total of 60 rehabilitation and 6 educational sessions. Patients of the CenterRehab program will participate in 24 supervised sessions and 36 home sessions, while the patients of the Home-Based program will participate in 2 supervised sessions and 58 home sessions, guided by telephone. After the 12-week period all participants will be recommended to continue practicing physical exercises at home or at a community center, and they will be invited for re-evaluation after 3 months. The outcomes will be evaluated at baseline, and after 3 and 6 months.

**Results:**

Participants are currently being recruited for the trial. Data collection is anticipated to be completed by October 2019.

**Conclusions:**

This is the first study in Brazil comparing the traditional cardiac rehabilitation approach with a novel, home-based protocol that uses an accessible and low-cost technology. If positive results are obtained, the study will contribute to establish a new and viable model of cardiac rehabilitation.

**Trial Registration:**

ClinicalTrials.gov NCT03605992; https://clinicaltrials.gov/ct2/show/NCT03605992

**International Registered Report Identifier (IRRID):**

DERR1-10.2196/13901

## Introduction

### Background

Cardiovascular diseases (CVDs), including its main form, the coronary artery disease (CAD), are the leading cause of death worldwide [1]. CAD has a negative impact on morbidity, quality of life, and survival of the population [2]. In Brazil, CAD is among the main causes of hospitalization and death [3]; thus, the implementation of programs that minimize these impacts is necessary [4-6].

The World Health Organization defined cardiac rehabilitation (CR) as a set of activities and interventions necessary to ensure the best physical, mental, and social conditions for the patients with chronic or postacute CVD, to be able to preserve or return to their appropriate place in the society by their own efforts [7]. The main goal of CR is the education and training for self-care, with an emphasis on physical exercise, as its positive effects on improving the quality of life as well as reducing hospitalization and the risk of death in CAD patients have been thoroughly demonstrated [8-10].

Despite the benefits of supervised CR programs, such interventions have been shown to be impractical in low- and middle-income countries (LMICs). Only 40% of them currently have a CR program, and in those, there is a grossly insufficient capacity [11]. In Brazil, it is estimated that more than 3.9 million people would benefit from a CR program; however, less than 20% of them have access to it [12]. The main obstacles for participation in CR programs in low-resource environments are the accessibility to programs provided by the public health system and the lack of time because of professional and family commitments and transport availability [10,12]. Therefore, alternative CR models are necessary to improve participation, considering the community diversities and the economic viability [13,14].

Therefore, the home CR model presents some advantages and improves patients’ participation and compliance to a healthy lifestyle and drug treatment as well as facilitates the patient and health professional education process [15,16].

Home-based CR is a term used to refer to CR at home or in other nonclinical settings, such as community centers, health clubs, and parks. This term also encompasses the use of information and communication technologies and hybrid form rehabilitation (ie, some sessions held in person at the rehabilitation clinic in conjunction with rehabilitation sessions at home) [16]. The home-based CR approach has been shown to be more efficient compared with conventional rehabilitation programs [16-19]. Studies showed that CR programs performed at home can overcome the traditional participation barriers and promote effects comparable with CR outpatients regarding the mortality, risk of recurrent coronary event, cardiovascular risk factors, and exercise capacity [17-19].

Different models of interventions and cardiac monitoring are used in home rehabilitation programs [5,6,15,17,19]. However, most models use equipment demanding a higher technological ability from the participants, such as manipulating digital equipment, downloading data from an exercise tracking equipment to a computer, using a chat software, and receiving information via email. Patients with a low education level living in a low-resource environment could have difficulties in making proper use of the required equipment. Thus, one of the challenges in designing a home-based CR program in LMICs is the use of a feasible, accessible, and viable technology for monitoring patients. Ideally, the technology should ensure an ideal heart rate range to allow for the expected training effects, without increasing the risk of adverse effects.

### Objectives

The aim of this trial is to verify the compliance and the effectiveness of a home-based CR program, which includes the health education and physical exercises components, mostly unsupervised and guided by telephone. We also compare this novel approach with the traditional, supervised CR program offered in most Brazilian hospital centers.

## Methods

### Study Design and Procedure

The study is a single-blind, randomized controlled trial. The researchers will be blinded for the treatment’s allocation during the duration of the trial. Owing to the nature of the intervention, neither the participants nor the program staff can be blinded to the allocation type. Patients will be recruited at the outpatient University Hospital's CR Center. The study will conform to the Consolidated Standards of Reporting Trials (CONSORT) guidelines for nonpharmacological interventions [20]. After being invited to participate in the study, the volunteers will sign a consent form and will be randomized into 2 different groups: traditional CR (mostly supervised) and home-based CR (mostly unsupervised). The randomization will be made in blocks of 4 volunteers at a time [21]. A blinded researcher will evaluate the participants before and after the intervention and will collect the data into a database.

**Figure 1:**
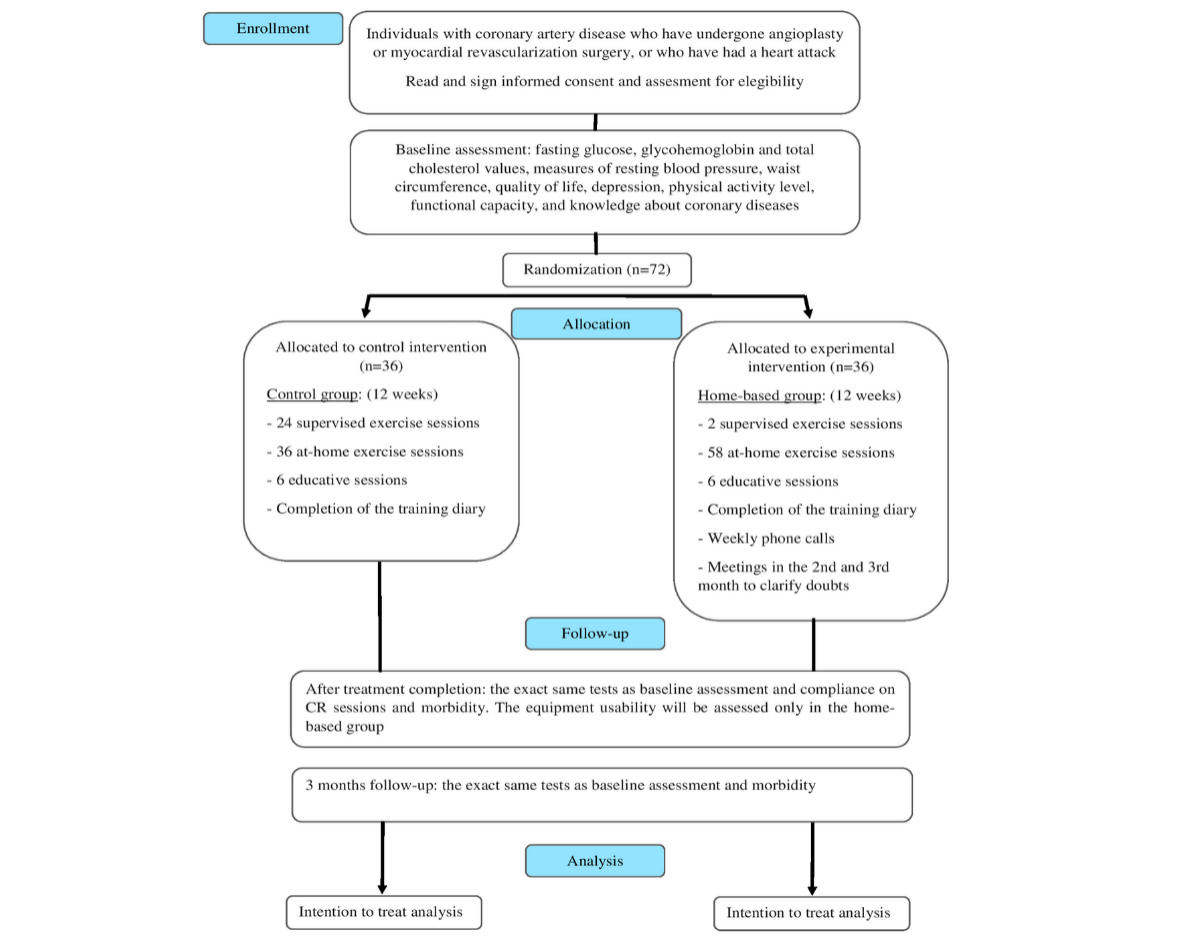
Consolidated Standards of Reporting Trials flow chart. CR: cardiac rehabilitation.

This study protocol is in accordance with the most recent version of the World Medical Association Declaration of Helsinki. This study was approved by the Ethics Committee in Research of the Universidade Federal de Minas Gerais, Brazil and was registered under the CAAE 51528615.3.0000.5149, on February 23, 2016. The CONSORT flowchart is reported in Figure 1.

### Patient and Public Involvement

Patients and the public were not directly involved in the study design. The intervention design was chosen based on previous studies, which reported that home-based CR programs promoted comparable effects with CR outpatient regarding the mortality, recurrent coronary event risk, cardiovascular risk factors, and exercise capacity [2,16-19,22]. The results of the study will be disseminated through institutional media, and educational activities will be conducted with CR patients and the study participants.

### Participants

Patients will be eligible if they meet the following inclusion criteria: (1) if they have a CAD and have undergone angioplasty or myocardial revascularization surgery and (2) if they had a heart attack, provided that they were considered at low and moderate risk for the practice of physical exercise of moderate intensity, according to the stratification for the risk of events during a cardiovascular rehabilitation program [23]. Moreover, the inclusion criteria would require that patients have clinical stability (according to a medical evaluation) and be resident of the Belo Horizonte metropolitan region. Participants will be excluded if the history of recent cardiac events or clinical decompensation is more recent than 1 month and if at least one of the following limitations is present: peripheral arterial occlusive disease, preventing the test of maximum exercise level (emergence of claudication before the maximal cardiorespiratory fatigue); chronic pulmonary disease (ie, chronic obstructive pulmonary disease, pulmonary fibrosis, and pulmonary arterial hypertension of precapillary etiology); history of ventricular fibrillation or sustained ventricular tachycardia in the last year; and physical, cognitive, and/or social limitations that prevent participation in a physical exercise program and the comprehension of monitoring the device usage.

### Intervention

The parameters for monitoring the exercise prescription compliance will be the same for both groups. An exercise session will be constituted by 5 to 10 min of warm up, 40 min of aerobic activity with a heart rate between 60% and 80% of the heart’s maximum rate, and 5 to 10 min of cool down [24]. The educational sessions will be given to both groups in 6 meetings of 40 min, using a systematized protocol [25]. In these meetings, topics regarding the control of risk factors and the treatment of CVDs will be taught. After the 12 weeks of intervention, all participants will be encouraged to continue practicing physical exercises either at home or at the community center and will be invited for a re-evaluation after 3 months.

### Groups

#### Traditional and Face-to-Face Cardiac Rehabilitation as Control Group

The control group will receive, in person, the usual program consisting of supervised exercises and health educational activities at the CR center. This intervention will last for 12 weeks, with a total of 60 sessions: 24 supervised and 36 home sessions (5 exercise sessions per week). The participants of this group will be instructed to complete a training diary, with information regarding the frequency and intensity of exercises (using a scale of perceived exertion) as well as the presence of symptoms during or after exercises. The same information will be registered in their individual file while exercising at the CR center.

#### Home-Based Cardiac Rehabilitation

The participants of this program will perform their exercise mostly at home. Weekly phone calls will be programmed to check the correct execution of the previous stage of the program and also identify and register the presence of symptoms and undesirable effects. Monthly meetings will be programmed for educational activities to verify if the exercises and training diary are being both performed and compiled correctly and to address any kind of issue by the participants.

This intervention will have a duration of 12 weeks, with a total of 60 sessions: 2 supervised sessions and 58 home sessions (5 exercise sessions per week).

During the first week, all individuals of the home-based group will be receiving training regarding the utilization of the monitoring equipment. A heart rate monitor with the heart rate zone individually calculated will be given to each participant at the first supervised session. Furthermore, all participants of this group will be using a step counter (pedometer) to monitor the number of prescribed exercises as well as an aid to compile the training diary with information regarding the frequency of exercises, the presence of symptoms during the exercise, the perceived exertion, and the number of daily steps.

### Measures

The participants will be invited to complete a sociodemographic questionnaire. The clinical characteristics will be extracted from the participant’s medical records, including the risk factors, cardiac history, results of cardiac examinations, comorbidities, and medications in use.

#### Primary Outcome: Compliance of Cardiac Rehabilitation Sessions

The primary outcome of this trial was carefully chosen. A correct exercise compliance promotes positive changes in behavior and lifestyle [18], besides reducing the risk for rehospitalization and improving quality of life [26].

The compliance on CR sessions will be analyzed by the percentage of participants who completed at least 75% (45/60) of the sessions. This parameter will be evaluated after 3 months.

#### Secondary Outcomes: Functional Capacity, Cost, Morbidity, Control of Risk Factors, Heart Health Behaviors, and Usability

Changes in the functional capacity will be analyzed using the incremental shuttle walk test (ISWT), a walking test that evaluates the functional capacity through the analysis of the walked distance [27].

The cost analysis and morbidity variables will be evaluated after 3 months and after 3 and 6 months, respectively, in both groups. The analysis of the programs’ cost will be made by calculating the total sum of each procedure in the 2 groups, considering the hospital’s payments table for procedures and services. Morbidity will be evaluated through a survey to identify the number of hospitalizations, complications, and the presence of adverse clinical events during the study period.

Resting blood pressure, measured in mm Hg, and waist circumference, measured in cm, will be analyzed. Waist circumference will be assessed at the superior border of the iliac crest, in accordance with the standardized guideline [28]. Alterations in fasting glucose (mg/dL), glycohemoglobin (%), and total cholesterol (mg/dL) values will be analyzed before the CR program and reanalyzed after 3 and 6 months.

Other behaviors will be assessed using psychometrically validated scales. Quality of life will be assessed using the Short Form 36 questionnaire [29], depression by the Patient Health Questionnaire-9 [30], the physical activity level using the Duke Activity Status Index Score [31], and the level of knowledge by the Coronary Artery Disease Education Questionnaire-Short Version [32].

Alterations in fasting glucose, glycohemoglobin, total cholesterol values, measures of resting blood pressure, waist circumference, quality of life, depression, physical activity level, functional capacity, and knowledge about coronary diseases will be evaluated at the beginning and after 3 and 6 months of the intervention in both groups. The assessment schedule is reported in Table 1.

The usability of the equipment used in the home-based group will be verified through the System Usability Scale after 3 months [33].

**Table 1:** Schedule of outcome assessments for control and home-based groups.

Outcome measures			Control group			Home-based group		
			Baseline	3 months	6 months	Baseline	3 months	6 months
**Primary outcome**								
	Compliance on cardiac rehabilitation sessions		—^a^	X^b^	—	—	X	—
**Secondary outcomes**								
	Functional capacity		X	X	X	X	X	X
	Cost analysis		—	X	—	—	X	—
	Morbidity		—	X	X	—	X	X
	**Risk factors**							
		Resting blood pressure	X	X	X	X	X	X
		Waist circumference	X	X	X	X	X	X
		Fasting glucose	X	X	X	X	X	X
		Glycohemoglobin	X	X	X	X	X	X
		Total cholesterol	X	X	X	X	X	X
	**Heart health behaviors**							
		Quality of life	X	X	X	X	X	X
		Depression	X	X	X	X	X	X
		Physical activity level	X	X	X	X	X	X
		Knowledge	X	X	X	X	X	X
	Usability		—	—	—	—	X	—

^a^No collection for the specified outcome measure on that date.

^b^Collection for the specified outcome measure on that date.

### Data Monitoring

An independent researcher, who will be blinded to the group allocation, will perform database management and the statistical analysis.

### Sample Size

On the basis of previous CR studies [34] and on our 7-year experience in providing rehabilitation services in a university hospital, we estimated a compliance of 70% in the control group and of 96% in the home-based group. We calculated that for an alpha significance level of 5% and a power of 80% in the Fisher exact *t* test, the sample size of each group should be at least 36 volunteers. We also calculated the sample size for the secondary outcome, the functional capacity (ISWT), considering an arbitrary small size effect of 0.25. To detect a statistically significant difference within and between groups (2×2), at least 34 volunteers in each group are needed, keeping the same values of alpha and power. After a pilot study with 10 subjects in each group, the sample calculation will be performed again. All the calculations were made using the G Power 3.1.9.2. software (Franz, Universitat Kiel) [35].

### Statistical Analyses

Data will be presented as a measure of the central tendency and dispersion. Data distribution will be analyzed using the Shapiro-Wilk test. All statistical analyses will be performed considering the intention-to-treat analysis and a per protocol basis to mitigate bias. The comparison between groups of the primary outcome will be made using the Fisher exact test, considering that the expected frequency of noncompliance of the home-based group may assume a value lower than 5%. Differences between groups and at the follow-up 3 and 6 months after the intervention, in addition to the interaction effect, will be analyzed using the generalized estimation equations [36]. In all models, the endpoint variable will be analyzed as a dependent variable and the variable at baseline and after 3 months as independent variables. An alpha value of 5% will be considered for statistical significance. Post hoc tests will be performed where significant differences are observed between the groups.

## Results

Recruitment started in February 2018 with an end date of October 2019. Between February 2018 and August 2019, 51 patients have consented to participate in this trial, and all of them were evaluated after 3 months. The recruitment was slower than estimated because of the reform of the CR sector during this period.

## Discussion

### Principal Findings

Various studies showed that home-based CR programs overcome the traditional participation barriers and promote effects comparable with CR outpatients regarding the mortality, risk of recurrent coronary event, cardiovascular risk factors, and exercise capacity [16-34]. These studies were mostly performed in the middle- and high-income countries, where the use of technologies is viable without great difficulties.

In LMICs, the use of technologies for CR programs is oftentimes not feasible. Thus, our study is important because it is performed in a public hospital in a country with a prevalence of low-income, low-schooling population, which have difficulties in using new technologies.

To our knowledge, this is the first study performed in Brazil that contemplates a remote CR program using an accessible and low-cost technology. If positive results are obtained, this study will contribute to establish a new model of remote CR, where the participants will be able to perform the prescribed exercises near home, at convenient times, minimizing the barriers for access to CR and reaching populations that frequently would not have accessibility to CR programs.

We believe that this study has a high potential to improve the care of patients affected by CAD in Brazil as well as in other countries from Latin America.

### Strengths and Limitation of This Study

An important strength of this study is the first to examining the potential of assessing CR remotely in Brazil using an accessible and low-cost technology. There is currently insufficient evidence on the effectiveness of home-based CR programs in LMICs, so the results may be used to improve the care of patients with coronary diseases in Brazil as well as in other LMICs. The limitation of this study is that it enrolls participants with low and moderate risk. Therefore, the results of this study may be used in the practice of cardiovascular rehabilitation within certain limitations.

## Abbreviations

CAD: coronary artery disease
CONSORT: Consolidated Standards of Reporting Trials
CR: cardiac rehabilitation
CVD: cardiovascular diseases
ISWT: incremental shuttle walk test
LMIC: low- and middle-income country
